# Risk factors for idiopathic granulomatous mastitis: scoping review

**DOI:** 10.1093/bjsopen/zrag137

**Published:** 2026-09-29

**Authors:** Rute S S Castelhano, Leah Argus, Daniel Ahari, Goonj Johri, Emma MacInnes, Shaneel Shah, Christopher Darlow, Vijay Sharma, Rudresh Shukla, Iain Lyburn, Nisha Sharma, Roisin Bradley, Cliona C Kirwan

**Affiliations:** Manchester University NHS Foundation Trust, Wythenshawe Hospital, Manchester, UK; Manchester University NHS Foundation Trust, Wythenshawe Hospital, Manchester, UK; Manchester University NHS Foundation Trust, Wythenshawe Hospital, Manchester, UK; Manchester University NHS Foundation Trust, Wythenshawe Hospital, Manchester, UK; Department of Surgery, Leeds Teaching Hospitals NHS Trust, UK; Manchester University NHS Foundation Trust, Wythenshawe Hospital, Manchester, UK; Antimicrobial Therapeutics and Pharmacodynamics, University of Liverpool, Liverpool, UK; Department of Infectious Diseases and Microbiology, Liverpool University Hospitals NHS Foundation Trust, Liverpool, UK; Department of Infectious Diseases and Microbiology, Liverpool University Hospitals NHS Foundation Trust, Liverpool, UK; Centre for Musculoskeletal Research, Faculty of Biology, Medicine & Health, University of Manchester, Manchester, UK; NIHR Manchester Biomedical Research Centre, Manchester University NHS Foundation Trust, Manchester, UK; Cobalt Medical Charity, Cheltenham, UK; Department of Radiology, Leeds Teaching Hospitals NHS Trust, UK; York and Scarborough Teaching Hospitals, NHS Foundation Trust, York, UK; Manchester University NHS Foundation Trust, Wythenshawe Hospital, Manchester, UK; Division of Cancer Sciences, School of Medical Sciences, Faculty of Biology, Medicine and Health, University of Manchester, Manchester, UK

**Keywords:** breast inflammation, reproductive history, lactation, hyperprolactinaemia, epidemiology, ethnicity

## Abstract

**Background:**

Idiopathic granulomatous mastitis (IGM) is a rare, benign, inflammatory breast condition. The diagnostic challenge can cause delays in diagnosis and treatment. The quality of evidence for the diagnosis and management of IGM is low. A scoping review of existing literature was conducted to determine risk factors for IGM, with the aim of informing clinical suspicion, earlier diagnosis, and treatment of this condition.

**Methods:**

A scoping review of the MEDLINE, Embase, and Cochrane Central Register of Controlled Trials (CENTRAL) databases (2003–2023) was performed following PRISMA-ScR guidance. Data on demographics, reproductive history, hormonal factors, body mass index (BMI), and smoking were extracted and subjectively compared to appropriate published population demographics of the relevant risk factors.

**Results:**

In all, 230 studies involving 13 062 patients were included. Most common countries of publication origin were Turkey (86 papers; 5233 patients), the USA (30 studies; 764 patients), and China (23 papers; 3990 patients), with three papers (25 patients) from the UK. Most common ethnicities reported were Hispanic (36%), Turkish (20%), White (9%), and Middle Eastern (8%). The mean age (34.7 years) was consistent across the literature, except for UK publications (mean age 50 years). High rates of history of pregnancy (> 90%) were reported, except for UK papers (68%); 64–85% of patients had a history of lactation; and the prevalence of hyperprolactinaemia (reported in 18% of papers) was 5% (*versus* 0.4% in the general population). BMI appears to match age and country-equivalent BMIs, with no association with diabetes. Smoking association is inconclusive.

**Conclusion:**

Risk factors for IGM appear centred on reproductive, hormonal, ethnicity, and possibly geographical factors, although the data are limited by inconsistent reporting, small study sizes, and retrospective study designs.

## Teaser

A scoping review of 230 studies (> 13 000 patients) found that idiopathic granulomatous mastitis is associated with reproductive and hormonal factors, particularly previous pregnancy, lactation, and hyperprolactinaemia, with possible ethnic and geographical influences. However, the evidence is limited by inconsistent reporting and predominantly retrospective studies.

## Introduction

Idiopathic granulomatous mastitis (IGM) is a benign chronic inflammatory condition of the breast, first described by Kessler and Wolloch in 1972^1^.

IGM usually presents as a tender, unilateral mass, with skin induration and swelling, sometimes with axillary lymphadenopathy, nipple retraction or *peau d’orange*^2^. The variable clinical and radiological presentation of IGM can mimic breast infection or breast cancer. Microbiological assessment is required to exclude infectious causes, and histopathological assessment is important, not just to rule out malignancy, non-granulomatous conditions, and non-idiopathic granulomatous mastitis, such as cystic neutrophilic granulomatous mastitis or xanthogranulomatous mastitis, but also to identify histopathological features associated with IGM^1,3^. Histologically, IGM is characterized by inflammatory breast changes, with non-caseating granulomas and multinucleate giant cells^4,5^. Fundamentally, the diagnosis of IGM is one of exclusion.

The described management of IGM is highly variable, ranging from using a period of watchful waiting to medical treatment, such as topical, intralesional, or systemic steroids and antibiotics, to radiological and surgical interventions. Conservative management may be an appropriate first-line approach, with the more interventional strategies reserved for refractory cases^6^. IGM has high relapse rates ranging from 5% to 50%, with reported rates of recurrence clustering around 20% overall^7^. IGM is a significant burden on patients (personally, socially, and economically) and healthcare resources.

Due to the nature of the diagnosis being one of exclusion, confirming IGM can be a diagnostic challenge for healthcare professionals. The differential diagnosis is broad, and specific tests are required to effectively rule out more diagnostically challenging differential diagnoses, including tuberculosis and cystic neutrophilic granulomatous mastitis^3^. The similarity of the clinical presentation to bacterial abscesses frequently leads to prolonged empiric antibiotic treatment before clinical suspicion is aroused. Limited awareness, and therefore the low index of suspicion of the presence of this rare condition, may also contribute to delays in diagnosis and the variability seen in management.

The quality of the evidence base for the diagnosis and management of IGM is low. As noted in a previous scoping review^8^, most studies (88%) are retrospective, with 65% of these being case series. Of published studies on IGM, 39% are from Turkey alone, with over three-quarters coming from Turkey, China, and Iran (79%)^8^. Only ten studies (including 76 patients) are from Europe^8,9^. The current literature may not reflect the clinical practice or potentially the disease course in a UK or equivalent population or healthcare system. The disproportionate volume of evidence from specific geographical regions and ethnicities may reflect a true higher incidence in these countries. It is also possible that this reflects overdiagnosis in healthcare systems where access to imaging and pathology may be less consistent, but equally it could reflect under-reporting or under-recognition, as a reflection of lack of clinical awareness, in other regions.

Fundamental to the diagnosis of IGM is clinical suspicion, based in part on a knowledge of risk factors. In recognition of the need to develop guidance for the diagnosis and management of IGM in a Western population and healthcare system, the Establish National Guidance for Idiopathic Granulomatous Mastitis (ENIGMA) collaborative group has been developed to determine the existing evidence base, the applicability of this evidence base to the UK population, and the gaps in the evidence base. The overarching aim of the ENIGMA programme of work is to develop guidelines for the diagnosis and management of IGM in the UK and similar populations and healthcare systems. National registries could support this, like the Granulomatous Mastitis Registry Study (GRAMAREG), which is a prospective registry and an ongoing European observational cohort study to evaluate risk factors and therapeutic strategies in patients with granulomatous mastitis^10^.

The aim of the present study was to analyse the evidence describing risk factors for IGM through a comprehensive scoping review of the existing literature. The reported risk factors will be considered in the context of the demographics of the relevant background population. This approach will then enable the contextualization of the available evidence to the UK, or other similar healthcare populations, where IGM is less frequently reported. Ultimately this analysis aims to provide greater clarity on the risk factors for IGM, thus facilitating improved clinical suspicion of this condition as a differential diagnosis. It will also identify gaps in the evidence base that require further research.

## Methods

An initial literature search on IGM revealed a high volume of publications with low-quality evidence more appropriate for a scoping, rather than a systematic, review. Scoping reviews were performed to identify and map the available evidence, identify gaps in knowledge, clarify concepts and, in this context, to provide insights for subsequent guideline development^11^. This review was conducted in line with PRISMA-ScR guidance^12^.

A search of the MEDLINE, Embase, and Cochrane Central Register of Controlled Trials (CENTRAL) databases was performed using the search term ‘(granulomat* or granulom*) AND (mastitis)’ within the title or abstract, including similar terms for records published between 2003 and 2023. This search was undertaken in conjunction with The Royal College of Surgeons of England Library and Archives Team (*supplementary methods*). Papers not published in English language, those describing animal studies or *in vitro* studies, conference abstracts, where full text was not available, and papers describing unlicensed treatments, such as Chinese traditional medicine and Ayurvedic medicine, were excluded. Records were stored and screened using the Rayyan® software (Rayyan Systems Inc., Cambridge, MA, USA)^13^; full details of the methodology are published elsewhere^8^.

All retrieved citations were initially screened by title and abstract by two independent reviewers (L.A. and S.S.), against a set of minimum inclusion criteria. In the event that consensus could not be achieved by the two reviewers, a third reviewer (C.K.) assessed the title and abstracts and determined whether the paper was suitable for inclusion. Subsequently, the full-text articles of all remaining papers were assessed by independent reviewers (L.A., G.J., R.C., S.C., S.S.) to determine whether they met the scoping review criteria.

Reverse citation searching of identified systematic reviews was undertaken to ensure the inclusion of relevant primary studies. However, the systematic reviews themselves were not included to avoid double counting of data. Studies focusing exclusively on histopathological or microbiological findings without reporting patient demographics were excluded. In addition, papers addressing cystic neutrophilic granulomatous mastitis, considered to represent secondary granulomatous mastitis related to *Corynebacterium* infection, were excluded.

Data collection was completed for the full-text records by the same five independent reviewers using a pro forma to include proposed risk factors and demographic data associated with IGM. The domains included were ethnicity, age, reproductive factors, medical co-morbidities, body mass index (BMI), smoking status, and oestrogen and prolactin concentrations. Data were collected following the Data Extraction for Complex Meta-Analysis (DECiMAL) guide^14^ and the Template for Intervention Description and Replication (TIDieR)^15^, and compiled using Microsoft^®^ Excel (Microsoft, Redmond, WA, USA) for validation and coding. Data were analysed using basic descriptive statistics.

Literature, including the grey literature, was searched to determine the best estimates of the age- and sex-matched (where possible) prevalence of proposed risk factors in the general population.

## Results

Of the 3208 abstracts initially identified, 230 papers met the scoping review criteria (*Fig. 1*). The total number of patients included in the scoping review was 13 062. As reported previously^8^, the studies were mainly from Turkey (86 papers; 5233 patients), USA (30 studies; 764 patients), and China (23 papers; 3990 patients), with only three studies from the UK^16–18^ (25 patients). The largest study sample size included 1309 patients from China, whereas the mean sample size was 58 patients (range 2–1309 patients)^8^.

**Figure 1:**
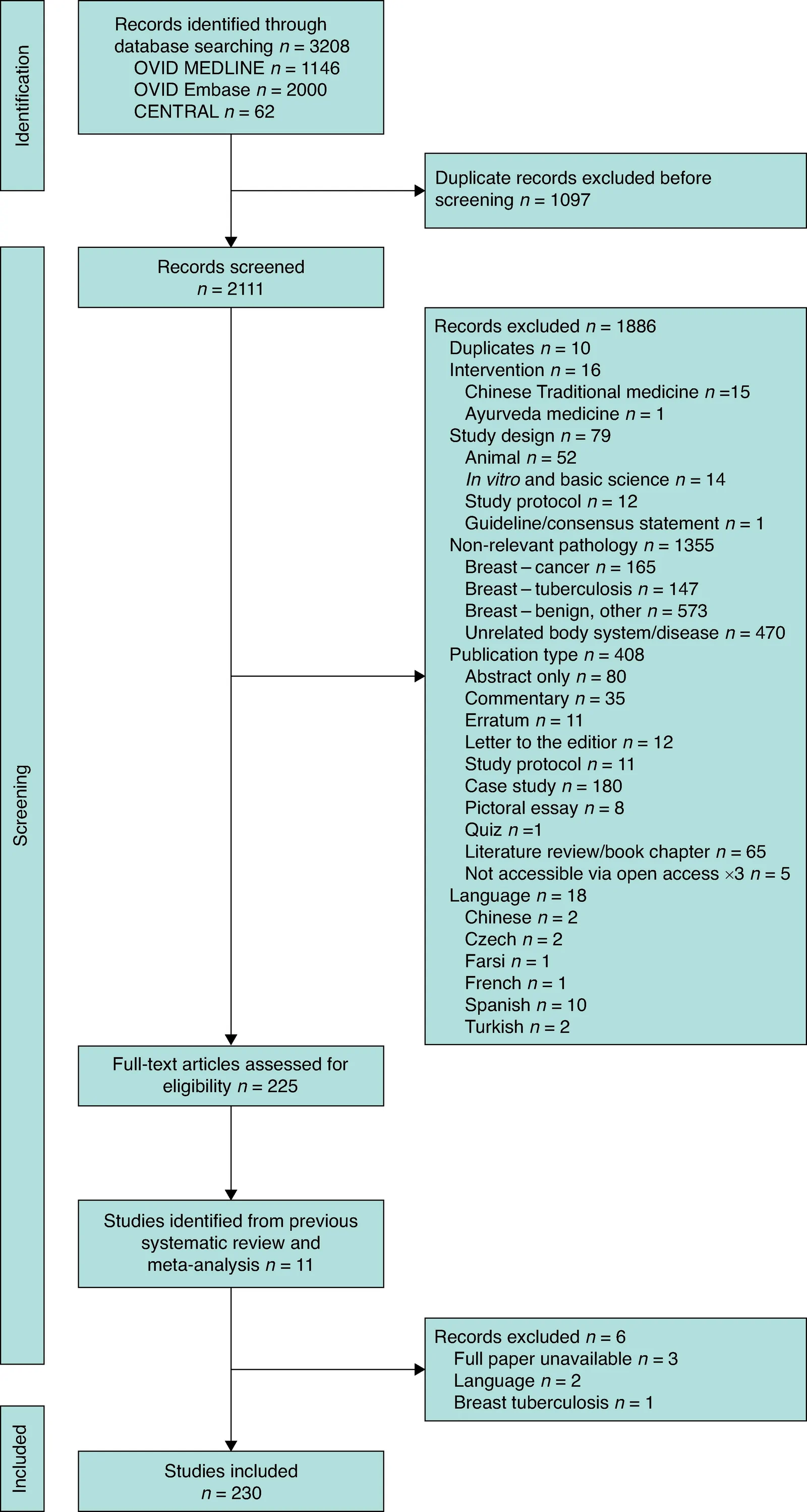
PRISMA flow diagram of scoping review In total, 3208 records were found through database searches and, after removing 1097 duplicates, 2111 titles and abstracts were screened. Most exclusions were due to irrelevant populations, interventions, study designs, publication types, or language. Of the 225 full-text articles assessed, all were initially considered eligible. In addition, backward citation searching of five eligible systematic reviews identified a further 11 papers, five of which were eligible for inclusion, bringing the total to 230. Overall, 225 were primary studies and five were secondary syntheses (four of which had meta-analyses). CENTRAL, Cochrane Central Register of Controlled Trials.

### Ethnicity

Ethnicity was reported in just 50 papers (22% of papers) including 1482 patients, corresponding to 11% of the scoping review patient population (*Table 1*). Of this combined cohort, the most common five ethnicities reported were Hispanic (36%), Turkish (20%), White (9%), Middle Eastern (8%), and Indian (5%).

**Table 1:** Ethnicity of the scoping review population

Ethnicity reported	No. of papers reporting on specific ethnicity	% Ethnicity represented in scoping review population
Hispanic	21	36.2%
Turkish	3	20.1%
White	23	9.3%
Middle Eastern	6	8.1%
Indian	6	5.5%
Chinese	5	4.7%
South East Asian	6	4.3%
Asian	13	3.4%
African American	12	2.4%
Other	12	2.4%
Black	6	1.2%
Maori	2	1.1%
Pacific Islander	2	1.0%
Hispanic American	2	0.1%
Indigenous American	2	0.1%

Of the 50 papers reporting ethnicity, 26 were from North America (USA and Canada) with 564 of 687 patients reported being Hispanic. Therefore, North America contributes the highest percentage of patients with ethnicity data in the literature (687 of 1482, 38%).

Of these studies from the USA, the ethnicity distribution in the IGM cohorts was as follows: Hispanic, 76%; White, 9%; Black, 7%; and Asian, 5%. This differs substantially from the ethnicity distribution of the background population in the USA, which is: White, 61%; Hispanic, 19%; Black, 12%; and Asian, 6%^19^.

Of the remaining 24 papers reporting ethnicity, three were from Turkey reporting 281 Turkish and three Middle Eastern patients (of 284 patients). Six papers from Europe (Cyprus, France, Netherlands, and the UK) reported ethnicity, with most patients being White (52 of 64, 81%).

In the two papers (22 patients) reporting ethnicity from the UK^16,18^, 14 patients (64%) were White, five (23%) were Asian, two (9%) were Black, and one (4%) was Middle Eastern. This compares to the England and Wales 2021 Census UK, where the total population was composed of 82% White, 9% Asian, and 4% Black^20^.

### Age

Age was reported in 180 papers (*Fig. 2*), with an overall mean age of 34.7 (range 11–87) years. The largest cohorts were from China (1309 patients), reporting a mean age of 31.3 years, followed by Iran (474 patients), with a mean age of 33.9 years, and Turkey (169 patients), with a mean age of 34 years. Interestingly, in the UK studies^16–18^, the mean age was 50 (range 47.5–52.3) years. Sex was mentioned in 202 papers (11 245 patients). Only four male patients were reported^21–23^. Menopausal status was reported in just 16 papers (1206 patients), with 168 women (14%) being post-menopausal.

**Figure 2:**
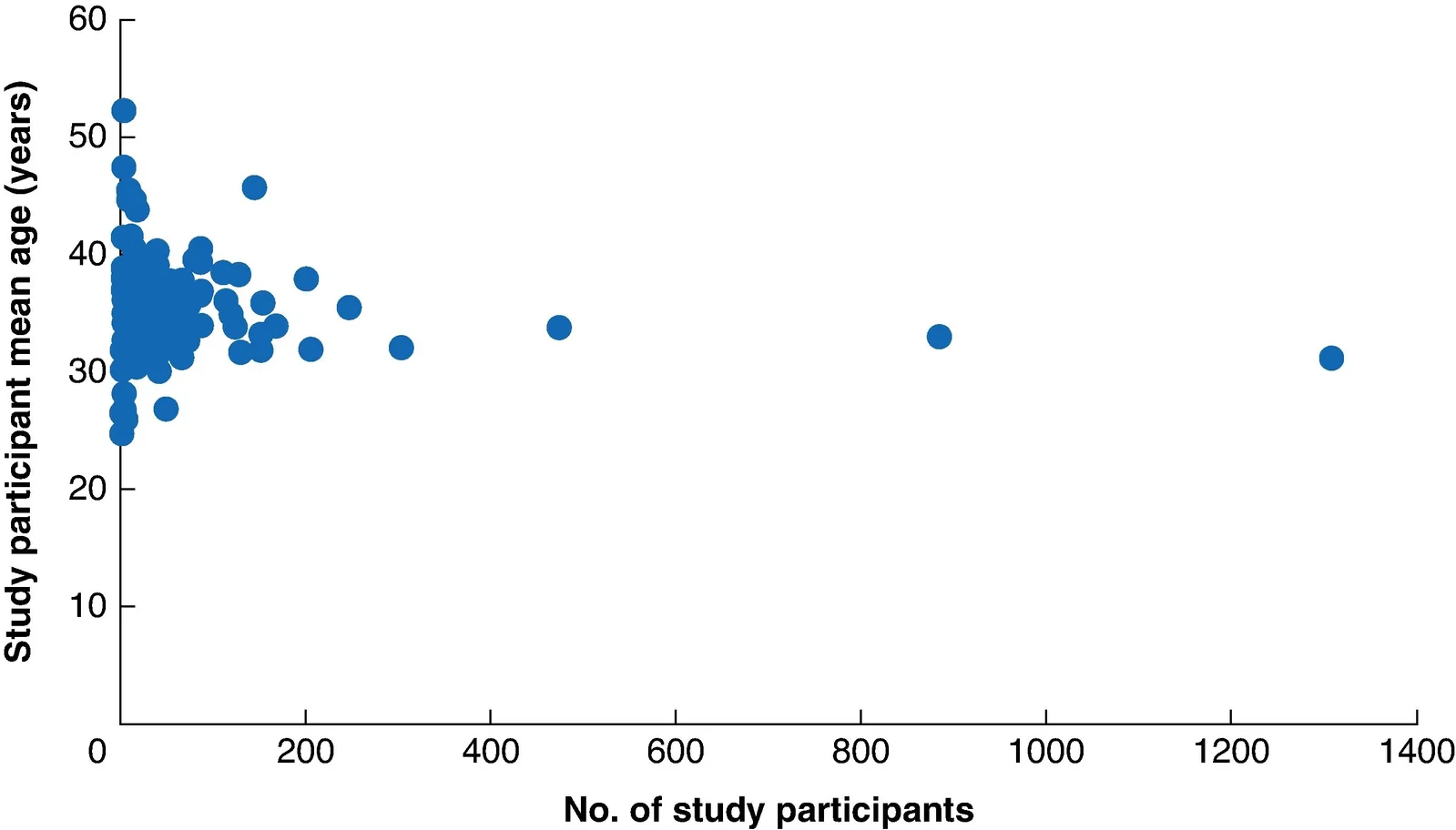
Study participant mean age plotted against study sample size

### Hormonal factors

#### Oral contraceptive use

The use of any oral contraception (OC) was reported in 75 papers (33%), which included a total of 4595 patients (35%). Of this patient cohort, 1016 (22%) reported current or previous use of OC. The largest cohort reporting on OC was Turkish^24^ (*n* = 720), with 94 patients (13%). The highest prevalence of OC use among patients with IGM was observed in Iran (36%), with the largest cohort investigating OC use being an Iranian paper of 474 women where 183 (39%) had a history of OC use^25^.

Of the 75 papers reported on the use of any OC, reported OC use ranged from 0% (in 11 studies; cohort size 2–206) to 100% (in 3 studies; cohort size 3–48). The time from the use of OC to symptoms was inconsistently reported.

#### Pregnancy

The presence of a history of pregnancy was reported in 95 papers, with 6257 of 6765 (92%) of this cohort reporting a previous or current history of pregnancy. Pregnancy at the onset of symptoms was reported in 45 papers, with 201 of 2793 (7%) women being pregnant at the time of symptom onset.

The two largest Chinese cohorts (with 1309 and 885 patients, respectively^26,27^) reported the 97% of women had a history of pregnancy. In these two Chinese cohorts, 6% of women were pregnant at the time of symptom onset. Similarly, high rates of a history of pregnancy were reported in the largest Turkish cohort^24^ (720 patients, with 92% having a history of pregnancy) and in the largest Iranian paper^25^ (474 patients, with 91% having history of pregnancy). One UK paper reported on pregnancy^17^, with 17 of 18 patients (94%) having a history of pregnancy. No papers consistently reported the time from pregnancy to the onset of symptoms.

Parity was specifically reported in 18 papers accounting for 471 patients; the mean was 2.4 children per woman, ranging from 2 to 9. Of these 18 papers, eight were from Turkey, with a mean parity of 3.4, and four were from the USA, with 2.1 children per woman. This compares to the national fertility rate in Turkey and in the USA of 1.9 and 1.7 children per woman, respectively^19^.

#### Lactation

A history of breastfeeding was reported in 80 papers by 4368 of 6812 patients (64%). Included in these were two large papers, one from a Chinese cohort^26^ (1309) reporting that 877 patients (67%) had history of lactation and one from Turkey^24^ (720) reporting that 612 patients (85%) had a history of lactation.

The onset of symptoms during lactation was reported in 17 papers, affecting 57 of 709 patients (8%). Of these 17 papers, 11 were from Turkey and Middle East (448 patients).

Only four papers^28–31^ (106 patients) reported on the time from the end of lactation to diagnosis, with a mean of 34 (range 24–43) months.

The length of time from completion of lactation was not reliably reported.

#### Hyperprolactinaemia

Hyperprolactinaemia was reported in 42 papers, affecting 366 of 4597 patients (7%). Eight cohorts were from Asia (seven from China, one from Hong Kong), with 301 of 1853 patients reported as having hyperprolactinaemia (16%), and six were from the Middle East, with 62 of 274 patients (23%) reported as having hyperprolactinaemia.

#### Prolactinoma and pituitary adenomas

Prolactinoma or pituitary adenomas were reported in 22 papers, affecting 25 of 939 patients (3%). Of these 22 papers, six (314 patients) were from North America (USA and Canada), with an overall reported prolactinaemia rate of 5%. Among the papers analysed, only one had a sample size > 100, which was from the USA^32^, reporting prolactinaemia in six of 120 patients (5%).

#### Antipsychotic use

The use of antipsychotics was reported in 88 of 2738 patients (3%) in 27 papers. Of these 27 papers, 11 were from Asia (seven from China; one each from Hong Kong, Singapore, Korea, and Japan) with 73 of 2214 patients reporting the use of antipsychotics (3%). Three small cohorts from Turkey (91 patients) reported antipsychotic use in four (4%) patients. Two small European cohorts (France and Netherlands) reported antipsychotic use in five patients of the combined cohort total of 26 patients (19%). The prevalence of antipsychotic use in the Chinese female population is reported to be low (1%), with its use higher in American and European female populations (14%)^33,34^.

### Body mass index

BMI was reported in 25 studies with a total of 2005 patients. Fifteen studies reported mean BMI, giving an overall mean BMI of 27.3 kg/m^2^ for the 1111 patients included in these studies. In nine papers (863 patients), only the weight category (that is, underweight, normal weight, overweight, and obese) was reported, with 43% of patients being overweight and 22% being obese (*Fig. 3*).

**Figure 3:**
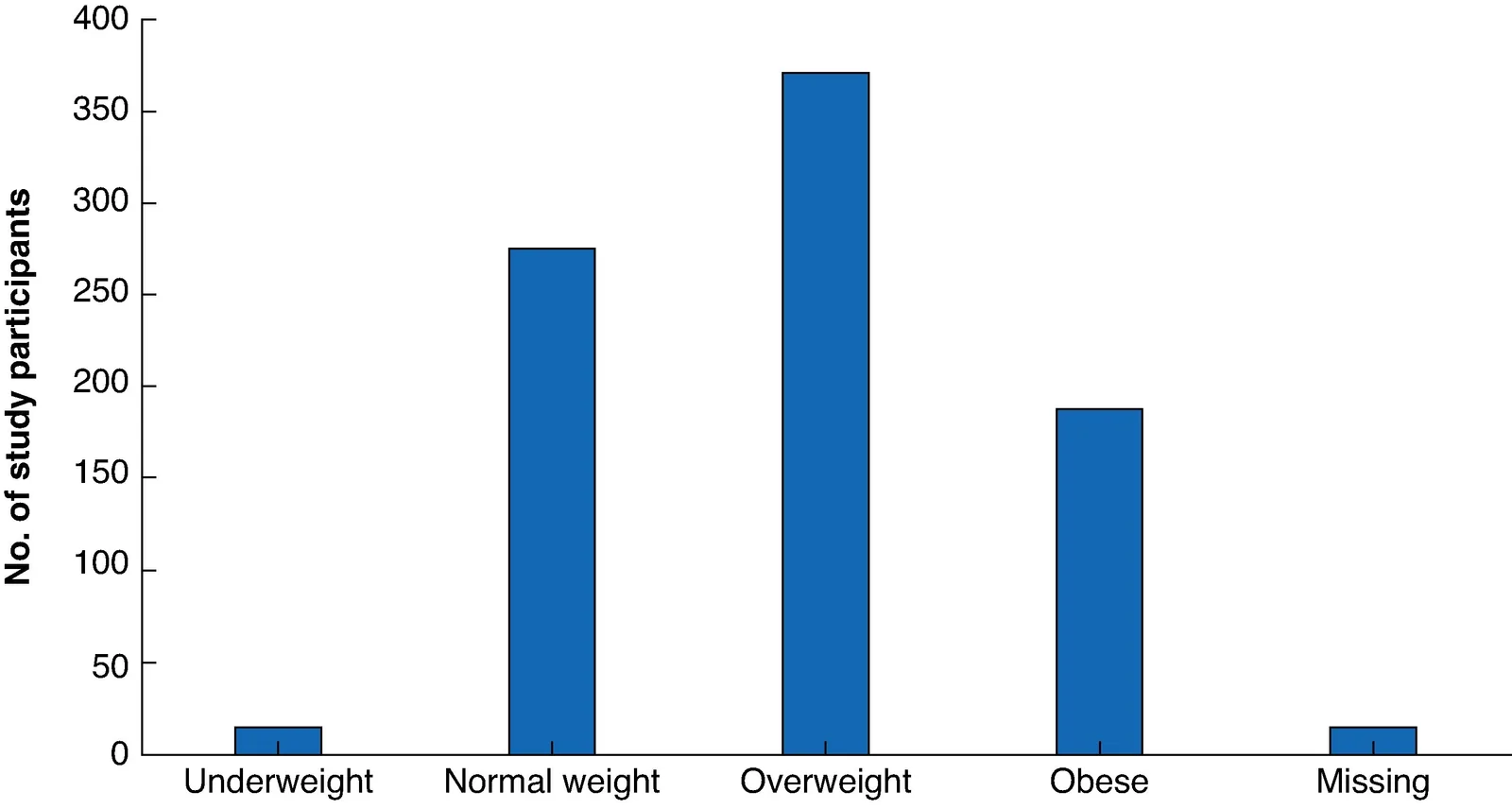
Number of participants per weight category Underweight: BMI < 18.5 Normal weight: BMI 18.5–24.9 Overweight: BMI 25.0–29.9 Obese: BMI >30.0

Six papers had a sample size of more than 100 patients: four papers from Turkey^35–38^ (530 patients), with a mean BMI of 26.5 kg/m^2^; one from the USA^32^ (120 patients), with a mean BMI of 30.1 kg/m^2^; and one from China^39^ (105 patients), with mean BMI of 24.1 kg/m^2^.

This BMI is broadly in keeping with country-matched population BMI for both age and sex; for example, in the USA, equivalent aged women in 2015–16 had mean BMI of 28.7 kg/m^2^, but the BMI of Hispanic women (19% of the North America population) was higher at 31.9 kg/m^240^. In Turkey, the mean BMI for those aged 30–34 years is 25.3 kg/m^2^, whereas that for people aged 35–39 years is 26.0 kg/m^241^. In China, overweight and obesity levels in women are relatively low, at 21.8% in 2013 and 29.9% in 2018^42^.

### Diabetes

Diabetes was described in 40 papers, affecting a total of 165 of 4477 patients (4%). Of the largest papers (> 100 patients) reporting on diabetes, five were from Turkey^24,36,43–45^, with 4% of patients affected, three were from China^26,39,46^, with 2% of patients affected, and one was from Iran^25^, also with 2% of patients affected.

### Smoking

Smoking was reported in 70 papers, as a factor for 638 of 3259 patients (20%). Of these papers, 36 were from Turkey, with 434 of 2050 patients (21%) reportedly smoking, nine were from the USA, with 27 of 299 patients (9%) smoking, three were from China^39,47,48^, with 103 of 248 patients (41%) smoking, and two were from the UK, with four of 22 patients (18%) smoking.

Among the four papers reporting on smoking with the largest cohorts (> 100 patients), the highest number of smokers was identified in the Turkish cohort: 133 of 597 patients (22%). This was followed by the USA (10 of 120 patients, 8%), China (89 of 105 patients, 85%), and then Hong Kong (5 of 102 patients, 5%). Comparing this with the current (2026) data on smoking for the general female population in different countries, China, Turkey, and the UK have significantly lower rates of smoking, at 1%, 20%, and 11%, respectively^49,50^.

### Thyroid disorders

Hypothyroidism was reported in 13 papers (619 patients), with eight patients (1%) being affected. Most of the papers reporting on hypothyroidism were smaller studies, with a median study size of 47 participants. Other thyroid disorders were reported in 17 papers (2259 patients), affecting 92 patients (4%).

Other conditions, such as autoimmune disorders, rheumatological diseases, hypertension, cardiovascular disease, and breast trauma, were reported, but less frequently.

The above findings are summarized in *Table 2*.

**Table 2:** Overview of demographic and clinical associations identified in the scoping review of IGM

Category	Finding	Interpretation
Ethnicity	Hispanic > Turkish/Middle Eastern > White > Asian	US cohorts: Hispanic 76.5%, White 9%, Black 7%, Asian 5% UK: White 64%, Asian 23%, Black 9%, Middle Eastern 4.5% Reporting inconsistent globally
Age	Mean age 34.7 years	Affects women in their reproductive years
Oral contraceptive use	22% reported use	Higher than general population
Pregnancy history	> 90% of women had a previous pregnancy	Suggests possible pregnancy-related risk; higher than expected for age-matched background population
Parity	Mean 2.4 children per woman	Higher than national averages, supporting potential association with number of childbirths
Lactation	Reported in most studies, but details limited	Insufficient data on duration or timing relative to IGM onset
Hyperprolactinaemia	7% of patients	Higher than general premenopausal population
Prolactinoma/pituitary adenomas	3% of patients	Higher than general population
Antipsychotic use	3% of patients	Higher than general population
Body mass index	Mean 27.3 kg/m^2^	Comparable to general population
Diabetes	4% of patients	Comparable to population prevalence
Smoking	20% of patients	Data biased to one population; findings inconsistent

The table summarizes the main demographic and clinical factors described in the literature on IGM. For each category, the key findings are presented alongside a brief interpretation, highlighting where evidence appears consistent, where associations remain uncertain, and where no clear link has been identified. IGM, idiopathic granulomatous mastitis.

## Discussion

This scoping review identified 230 papers including 13 062 patients, most of which were from middle-income countries. This raises the question of whether ethnicity or geography and environmental factors are responsible for the possible increased prevalence of IGM^9^. The greatest number of publications in this scoping review came from Turkey, followed by the USA and then China. The greatest proportion of patients came from China (30%), Turkey (18%), and Iran (8%). However, whether this reflects the true global prevalence of IGM and a greater prevalence in these countries, underdiagnosis, a lack of awareness in other countries, or simply publication bias is not possible to determine.

In terms of ethnicity, this review highlights that IGM is more commonly reported in patients of Hispanic ethnicity, followed by Turkish, White, and Middle Eastern ethnicity. However, ethnicity data are not consistently reported. In the USA, ethnicity data have been more reliably reported than other countries since the US Food and Drug Administration (FDA) developed guidance and recommendations on a standardized approach for collecting and reporting on race and ethnicity in 2005^51^. Compared with the general population, the prevalence of Hispanic ethnicity in the IGM population is markedly higher (76% *versus* 19%) and the prevalence of White ethnicity is lower (9% *versus* 61%)^52^.

It is widely perceived that the incidence of IGM is higher among women from the Middle East^4,21,29,53^ and Asia^54,55^. The overall incidence of 27% reported in Turkish/Middle Eastern patients may simply reflect publication bias or may be a true higher incidence. The papers from the UK^16–18^, which reported ethnicity, indicate that those of White ethnicity are the most affected (64%), followed by those of Asian (23%), Black (9%) and Middle Eastern (4%) ethnicity. The UK IGM data support a higher incidence of IGM among Asian individuals, therefore a multiethnic disease, with 23% of patients with IGM being Asian compared with 9% of the general population being Asian; however, clearly, numbers are limited.

IGM frequently affects women in their reproductive years, with a mean age at presentation of 34.7 (range 11–87) years^6,56^. However, the aetiological factors underpinning this association remain unclear^57^.

OC usage has been identified in many studies as a potential risk factor for IGM^6,9,57^ and recurrent IGM^2^, with the pathogenesis being proposed to be related to high levels of exogenous oestrogen. In the cohort of patients in this scoping review for whom data on OC use was collected, 22% of patients reported using OC. The highest prevalence of OC use among patients with IGM was observed in Iran (36%); this compares to 18% of the general female population of reproductive age in Iran using OCs^58,59^. The largest cohort reporting on OC use was from Turkey, with 13% of patients (94 of 720) using OC. This correlates with a questionnaire-based study^60^ designed to estimate the distribution of contraceptive use among women of reproductive age in a Turkish population, which found that 11% of Turkish women reported a history of using any type of OC. This may suggest an association between OC use and an increased risk of developing IGM; however, it could also reflect the difference between current and ‘current and previous use’ (the definition commonly used in IGM papers).

High levels of oestrogen and/or progesterone have been hypothesized as relevant in the pathogenesis of IGM. Uysal *et al*.^27^ and Omranipour *et al*.^29^ have described a statistically significant association between a history of pregnancy and IGM. The frequency of reporting a history of pregnancy highlights the perception that IGM may be a pregnancy-associated disease. It is interesting that the mean age of disease onset in the Chinese cohort is 31.3 years, with 96% of women having a history of pregnancy, which compares to a mean age of first child of 28.5 years^61^ and a childlessness rate of 5%^62,63^. These findings are similar for Iran, where the mean age of disease onset is 34 years, with 91% of the women having history of pregnancy, comparing with a mean age of first child of 29 years and a childlessness rate of 8%^64^. Given childlessness rates are reported at the end of the fertile age range (approximate age 50 years), the finding of similar ‘history of pregnancy rates’ in the younger cohort of IGM patients, compared with the background population ‘non-childless’ rate, highlights a possible association with pregnancy (beyond the fact that IGM is most common in women of reproductive age). Interestingly, rates of childlessness in China were reported to be 5% in 2020 population census, having increased from 1% in the 1990 Census^65^, with unofficial statistics reporting 8% of married Iranian women are childless^64^, highlighting similar rates of a history of reported pregnancy in the background population^64,65^.

Parity has also been considered in the aetiology of IGM due to the inherent hormonal changes^66,67^. In the present study, the mean parity was 2.4 children per woman. Most papers were from Turkey (8) with a mean parity of 3.4 children per woman. Four papers were from the USA^38^, with a mean parity of 2.1 children per woman^68–70^. According to the latest statistical data^71^, the national fertility rate in Turkey is 1.9 children per woman, compared with 1.7 children per woman in the USA. This demonstrates higher parity in patients with IGM, supporting a possible association with parity.

Taking into consideration that IGM frequently affects women in their reproductive years and those with higher parity, a history of lactation is expected to be present in this cohort of patients. Therefore, a limitation has been identified because most papers only report on history of lactation and not specifically about the length of time from completion of lactation to diagnosis or current lactation status during the development of IGM. Some studies describe IGM during breastfeeding as uncommon^6,32^; however, further information regarding lactation is required to determine the association between lactation and the development of IGM.

Hyperprolactinaemia can be induced by either physiological conditions, such as pregnancy, lactation, stress or physical exertion, or by pathological conditions, including drug-induced causes, liver cirrhosis, and hormonal dysregulation such as prolactinomas and pituitary adenomas^72^. The prevalence of hyperprolactinaemia in the healthy premenopausal population has been reported to be 4%^73^. However, in this review, the prevalence of hyperprolactinaemia was 7%, more frequently reported in cohorts from China and Hong Kong (16%). The prevalence of hyperprolactinaemia in Chinese premenopausal patients with breast conditions is reported to be 36%^74^, which is significantly higher than in the healthy premenopausal population. The prevalence of hyperprolactinaemia ranges from 0.4% in an unselected normal UK adult population to 4% in a healthy premenopausal Spanish population^75^. This scoping review suggests that hyperprolactinaemia may be higher among patients with IGM in than the general population, suggesting a possible link between high levels of prolactin and the development of IGM.

Similarly, prolactinoma and pituitary adenomas have been described as contributing factors for IGM due to high levels of prolactin associated with these conditions^76^. In the present review, prolactinoma and pituitary adenomas were reported for 3% of patients with IGM, with the highest incidence reported in studies from North America (5%). In North America, the mean prevalence of prolactinoma is estimated to be 30 per 100 000 women, with a peak prevalence in women aged 25–34 years^77^. Similarly, in China, the incidence of prolactinoma in the female population peaks around the age of 34 years and gradually declines after the age of 35 years^74^. These findings could also explain why the incidence of hyperprolactinaemia in premenopausal women is significantly higher.

Dopamine normally inhibits the secretion of prolactin from the pituitary gland and antipsychotic drugs are thought to interfere with this inhibitory effect, resulting in elevated prolactin levels; thus, an association between antipsychotic drugs and IGM has been proposed^78^. The finding of rates of antipsychotic drug use in Asian and European patients of 3% and 19% (*versus* 1% and 14%, respectively, in general populations) suggests a higher prevalence of antipsychotic drug use among patients with IGM than in the general population^33,34^.

A high BMI is commonly reported as a potential risk factor for IGM, because obesity has been shown to be associated with increased oestrogen production from adipose tissue, which has been proposed to play a role in the development of IGM^79,80^. In this scoping review, the mean BMI for the population with IGM was very similar to that of the general population, which is at odds with the previously reported association between IGM and BMI^29^. However, this comparison is limited by accurately time-matching population-level BMI data, given the global increase in obesity with time.

Diabetes was the most commonly reported co-morbidity in this review, affecting 4% of patients with IGM. Turkish IGM cohorts had the highest rate of diabetes (4%), followed by China (2%) and Iran (2%); this compares to a prevalence of diabetes of 3% in women aged 30–34 years in Turkey^81^, suggesting diabetes is not a risk factor for IGM.

In the 70 papers reporting the incidence of smoking, 20% of IGM patients were current smokers. This is much higher than current smoking prevalence. However smoking is on the decline, and the data collected on these cohorts spans a time period of 2003–2023. In China, the smoking rate among women has decreased since the early 2000s. In 2005, the rate was at 2.7%, decreasing to 2.4% in 2010 and to 1.4% currently^82^. A similar trend can be found among women in the UK, where the smoking rate was at 23% in 2005, decreasing to 20% in 2010 and now being at 10.7%^83^. This highlights the challenge of accurately matching populations.

This study has some limitations. The greatest number of publications included in the review came from Turkey, the USA, and China. Data from some regions, particularly Europe (except Turkey), are under-represented which may represent geographical and reporting bias. In addition, certain ethnic groups, such as Hispanic and Turkish populations, have a high prevalence of IGM that may reflect its true distribution or be an overrepresentation due to geographical publication bias.

This scoping review largely consists of case series and cohort studies, with the majority being retrospective^7,8^. A high number of studies had a small sample size. This may lead to inconsistencies in the findings and limits their reliability. Where possible, steps were taken to mitigate these issues. Single case reports were excluded to avoid relying on very limited evidence. However, small case series (≥ 20 patients) were kept; because IGM is such a rare condition, excluding them altogether would risk losing important insights from countries or centres that only occasionally encounter the disease. Removing them would also bias the review towards regions with higher reporting rates.

Most studies included in this review were from single centres, which limits the ability to draw conclusions that apply to a broader population. The predominance of retrospective data also raises the possibility of overlapping cohorts and a lack of reliability of the data, and is, therefore, a risk of reporting bias.

Many studies provided limited demographic or ethnicity data, making it difficult to assess the influence of these factors. Important factors, such as BMI and lactation history, were also frequently unreported, limiting the ability to fully understand their impact. Similarly, data regarding OC use may vary between countries, because access to hormonal contraception and methods of documentation vary considerably between healthcare systems and geographic regions. In settings where OCs are obtained without prescription or through informal routes, use may be incompletely captured within medical records, introducing the potential for under-reporting and limiting the comparability of reported rates between populations. A major challenge is the absence of standardized diagnostic criteria for IGM, which undoubtedly contributes to the differences in reported prevalence across studies.

Despite these limitations, this review offers relevant information on IGM that allows a comprehensive understanding of the disease from multiple populations and their healthcare systems.

The data from this scoping review of a combined cohort of 13 062 patients suggests IGM predominantly affects women of reproductive age, peaking at age 34 years. Rates appear higher in Hispanic and Asian women, but the high reporting in Turkish women is difficult to differentiate between reporting bias and true increased risk due to the lack of comparative data. There appears to be an association with previous pregnancy and higher parity. OC use, hyperprolactinaemia, and antipsychotic drug use appear more common in IGM populations than rates in broadly matched populations, supporting a possible prolactin-related association. BMI, diabetes, and smoking did not show clear associations with IGM. Overall, the findings suggested a multifactorial aetiology centred on reproductive, hormonal, and possibly geographic factors, although interpretation is limited by inconsistent reporting, small study sizes, and retrospective study designs.

There is a need for more structured approaches to improve consistency in the diagnosis and management of IGM in the setting of limited high-quality evidence. This may be supported by the development of standardized diagnostic pathways and treatment algorithms based on evolving data and expert consensus. Alongside this, national and international registries would provide a valuable platform for future research, prospective studies, and clinical trials, as well as enabling ongoing refinement and audit of emerging guidance. In this context, the GRAMAREG aims to better characterize disease presentation, treatment response and outcomes, including imaging features and recurrence^10^. Without coordinated efforts of this kind, variability in practice and delays in the diagnosis of IGM are likely to continue.

## Supplementary Material

zrag137_Supplementary_Data

## Data Availability

Data are available upon reasonable request.
