# Supplementary material for: Risk factors for idiopathic granulomatous mastitis: scoping review
Source: BJS Open. 2026 Sep 29;10(5):zrag137. doi: 10.1093/bjsopen/zrag137 (PMC13623002; doi:10.1093/bjsopen/zrag137)
Supplement: Supplementary file 1 [file zrag137_supplementary_data.docx]

**Risk factors for Idiopathic Granulomatous Mastitis: scoping review**

**Authors:**
Rute S S Castelhano^1^, Leah Argus^1^, Daniel Ahari^1^, Goonj Johri^1^, Emma MacInnes^2^, Shaneel Shah^1^, Christopher Darlow^3,4^, Vijay Sharma^4^, Rudresh Shukla^5^, Iain Lyburn^6^, Nisha Sharma^2^, Roisin Bradley^7^, Cliona C Kirwan^1,5^

**Author Affiliations:**

1. The Nightingale Centre (Manchester University NHS Foundation Trust), Wythenshawe Hospital, Southmoor Road, M23 9LT, Manchester, UK
2. Leeds Teaching Hospitals NHS Trust, UK
3. Antimicrobial Therapeutics and Pharmacodynamics, University of Liverpool, Liverpool, UK
4. Liverpool University Hospitals NHS Foundation Trust, Liverpool, UK
5. Division of Cancer Sciences, School of Medical Sciences, Faculty of Biology, Medicine and Health, University of Manchester, UK
6. Cobalt Medical Charity, GL53 7AS, Cheltenham, UK
7. York and Scarborough Teaching Hospitals, NHS Foundation Trust, UK

***Corresponding author:*** Professor Cliona C Kirwan

**Email:** Cliona.Kirwan@manchester.ac.uk
**Address:** The Nightingale Centre, Manchester University Foundation Trust, Southmoor Road, Wythenshawe, Manchester, M23 9LT
**Tel:** 0161 291 3534

**Category in which manuscript is being submitted:** Review article

**Funding:**

The ENIGMA Delphi consensus meeting was supported by the Association of Breast Surgeons (UK) Research Development Grant to the value of £9600.

C. C. K. is supported by a Masonic Charitable Foundation Fellowship (Royal College of Surgeons/Masonic Charitable Foundation Chair of Surgical Trials grant of £500 000).

I.L. is partially supported by Cobalt Medical Charity, Cheltenham.

**Conflict of Interest Statement:** We declare no conflict of interest.

**Data Availability Statement:** Data are available upon reasonable request.

**Ethical approval:** Ethical approval not required

**Supplementary Methods**: Search strategy for Ovid MEDLINE(R) electronic database

| Search Number | Search Terms |
| --- | --- |
| 1 | granulomatous mastitis/ |
| 2 | (granuloma* adj4 (mastitis or mastodynia or mastalgia)).ti,ab,kw,kf. |
| 3 | ((lobular or lobar) adj4 (mastitis or mastodynia or mastalgia)).ti,ab,kw,kf. |
| 4 | (cystic neutrophilic adj4 (mastitis or mastodynia or mastalgia)).ti,ab,kw,kf. |
| 5 | ((nonpuerperal or "non-puerperal" or nonlactation* or "non-lactation*" or periductal) adj4 (mastitis or mastodynia or mastalgia)).ti,ab,kw,kf. |
| 6 | 1 or 2 or 3 or 4 or 5 |
| 7 | breast/ or mammary glands, human/ or nipples/ or (breast* or nipple* or areola* or "milk duct*" or "milk gland*").ti,ab,kw,kf. |
| 8 | granulomatous mastitis/ or granuloma*.ti,ab,kw,kf. |
| 9 | 7 and 8 |
| 10 | 6 or 9 |
| 11 | limit 10 to last 20 years |
| 12 | limit 11 to english language |

Table A2: Search strategy for Embase electronic database

| Search Number | Search Terms |
| --- | --- |
| 1 | granulomatous mastitis/ |
| 2 | (granuloma* adj4 (mastitis or mastodynia or mastalgia)).ti,ab,kw,kf. |
| 3 | ((lobular or lobar) adj4 (mastitis or mastodynia or mastalgia)).ti,ab,kw,kf. |
| 4 | (cystic neutrophilic adj4 (mastitis or mastodynia or mastalgia)).ti,ab,kw,kf. |
| 5 | ((nonpuerperal or "non-puerperal" or nonlactation* or "non-lactation*" or periductal) adj4 (mastitis or mastodynia or mastalgia)).ti,ab,kw,kf. |
| 6 | 1 or 2 or 3 or 4 or 5 |
| 7 | exp breast/ or breast abscess/ or (breast* or nipple* or areola* or "milk duct*" or "milk gland*").ti,ab,kw,kf. |
| 8 | granulomatous mastitis/ or granuloma*.ti,ab,kw,kf. |
| 9 | 7 and 8 |
| 10 | 6 or 9 |
| 11 | limit 10 to last 20 years |
| 12 | limit 11 to english language |

Table A3: Search strategy for Cochrane Central Register of Controlled Trials (CENTRAL) and Cochrane Database of Systematic Reviews (CDSR) electronic database

| Search Number | Search Terms |
| --- | --- |
| 1 | MeSH descriptor: [Granulomatous Mastitis] explode all trees |
| 2 | ((granuloma*) NEAR/4 (mastitis or mastodynia or mastalgia)):ti,ab,kw |
| 3 | ((lobular or lobar) NEAR/4 (mastitis or mastodynia or mastalgia)):ti,ab,kw |
| 4 | ((cystic neutrophilic) NEAR/4 (mastitis or mastodynia or mastalgia)):ti,ab,kw |
| 5 | ((nonpuerperal or "non-puerperal" or nonlactation* or "non-lactation*" or periductal) NEAR/4 (mastitis or mastodynia or mastalgia)):ti,ab,kw |
| 6 | #1 OR #2 OR #3 OR #4 OR #5 |
| 7 | MeSH descriptor: [Breast] explode all trees |
| 8 | ((breast* or nipple* or areola* or "milk duct*" or "milk gland*")):ti,ab,kw |
| 9 | #7 OR #8 |
| 10 | MeSH descriptor: [Granulomatous Mastitis] explode all trees |
| 11 | (granuloma*):ti,ab,kw |
| 12 | #10 OR #11 |
| 13 | #9 AND #12 |
| 14 | #13 OR #6 |
| 15 | #13 OR #6 with Cochrane Library publication date Between Jan 2003 and Dec 2023 |

Table A4: Summary of Scoping Review Search

| Category | Item(s) |
| --- | --- |
| Search title | Scoping review of idiopathic granulomatous mastitis: Clinical management and research methodology |
| Population(s) | All patients, all age groups  All stages of management - clinical suspicion, diagnosis, management, follow-up |
| Intervention(s) | All interventions including: surveillance, medical management, surgical management |
| Outcomes | Incidence and prevalence  Diagnostic criteria  Management |
| Exclusion criteria | Not in the English language  Animal studies  In vitro studies  Cadaveric studies  Conference abstracts  Full-text not available  Non-original research (e.g., response/comment, letter to the editor, commentary, literature/narrative/scoping review, opinion piece)  Trial or study protocol  Non-idiopathic granulomatous mastitis (i.e., tuberculosis, sarcoidosis, IgG4-related disease) – with the exception of cystic neutrophilic granulomatous mastitis  Intervention not licensed by Medicines and Healthcare Products Regulatory Authority (MHRA) (e.g., Ayuvredic or Traditional Chinese Medicine therapies) |
| Search period | 2003 - 2023 |
| Comments | Initial sift (study design only) conducted by Information Specialist |
